# The genome sequence of barren brome, *Bromus sterilis* L. (Poaceae)

**DOI:** 10.12688/wellcomeopenres.22994.2

**Published:** 2024-11-20

**Authors:** Maarten J. M. Christenhusz, Royal Botanic Gardens Kew Genome Acquisition Lab, Plant Genome Sizing collective, Darwin Tree of Life Barcoding collective, Wellcome Sanger Institute Tree of Life Management, Samples and Laboratory team, Wellcome Sanger Institute Scientific Operations: Sequencing Operations, Wellcome Sanger Institute Tree of Life Core Informatics team, Tree of Life Core Informatics collective, Darwin Tree of Life Consortium

**Affiliations:** 1 Royal Botanic Gardens Kew, Richmond, England, UK; 2 Curtin University, Perth, Western Australia, Australia

**Keywords:** Bromus sterilis, barren brome, genome sequence, chromosomal, Poales

## Abstract

We present a genome assembly from an individual *Bromus sterilis* (the barren brome; Streptophyta; Magnoliopsida; Poales; Poaceae). The genome sequence has a total length of 2,677.90 megabases. Most of the assembly is scaffolded into 7 chromosomal pseudomolecules. The mitochondrial and plastid genome assemblies have lengths of 523.28 kilobases and 136.96 kilobases, respectively. Gene annotation of this assembly on Ensembl identified 29,147 protein-coding genes.

## Species taxonomy

Eukaryota; Viridiplantae; Streptophyta; Streptophytina; Embryophyta; Tracheophyta; Euphyllophyta; Spermatophyta; Magnoliopsida; Mesangiospermae; Liliopsida; Petrosaviidae; commelinids; Poales; Poaceae; BOP clade; Pooideae; Triticodae; Bromeae; *Bromus*; *Bromus sterilis* L., 1753 (NCBI:txid55777).

## Background

**Figure 1.**
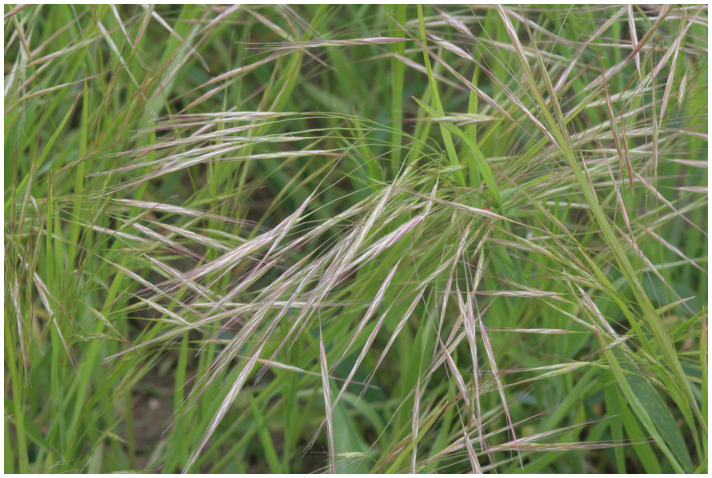
Photograph of *Bromus sterilis* (not the specimen used for genome sequencing). Image by HermannSchachner.

Barren brome, *Bromus sterilis* L. ( Figure 1), is a common grass of disturbed ground, dry hedge banks and roadsides. It is native to central and southern Europe and the Mediterranean Region east to Kyrgyzstan. It is widely naturalised in Britain, Ireland, the Baltic Region, East Asia, southeastern Australia, New Zealand, North America and southern South America ( POWO, 2024). Often dispersed through farming as a weed of wheat crops, it and can cause serious harm to the crop yield and biodiversity of field margins ( Lintell Smith *et al.*, 1999; Marshall, 2009).

*Bromus sterilis*, also sometimes treated as *Anisantha sterilis* (L.) Nevski, is an annual grass with soft culms and loose, drooping paniculate inflorescences with rough branches that are much longer than the spikelet. Spikelets are compressed, V-shaped with long awns, usually turning purplish when ripe. Flowers are pollinated by wind, and thus its pollen can contribute to allergies in urban areas. Seeds germinate in autumn and the plant flowers in mid spring.

*Bromus sterilis* is primarily selfing and largely inbred, with little intrapopulation variation at the genetic level. Microsatellite studies have shown that *B. sterilis* exists as numerous separate and genetically different lines, which are maintained by inbreeding, but which occasionally outcross ( Green *et al.*, 2001). Some UK populations have reduced glyphosate sensitivity and appear to be in the process of evolving resistance ( Davies *et al.*, 2019; Davies *et al.*, 2020).

While both diploid (2 *n* = 2 *x* = 14) and tetraploid (2 *n* = 4 *x* = 28) individuals have been reported in the literature (e.g. Oja & Laarmann, 2002), so far, the chromosome counts from UK-sourced material have been diploid (e.g. Montgomery *et al.*, 1997).

For this project, we sampled the specimen from rough ground in the Royal Botanic Garden, Kew. This high-quality genome will help in understanding the population dynamics, its response to climate change and the evolution of pesticide resistance in this species.

## Genome sequence report

The genome was sequenced from a specimen of *Bromus sterilis* ( Figure 1) collected from the Royal Botanic Gardens Kew, Richmond, UK (latitude 51.47, longitude –0.30). Using flow cytometry, the genome size (1C-value) was estimated to be 3.20 pg, equivalent to 3,120 Mb. The genome was sequenced using Pacific Biosciences single-molecule HiFi long reads, generating a total of 78.79 Gb (gigabases) from 8.43 million reads. The coverage was estimated as 27-fold, based on the GenomeScope size estimate.

**Figure 2.**
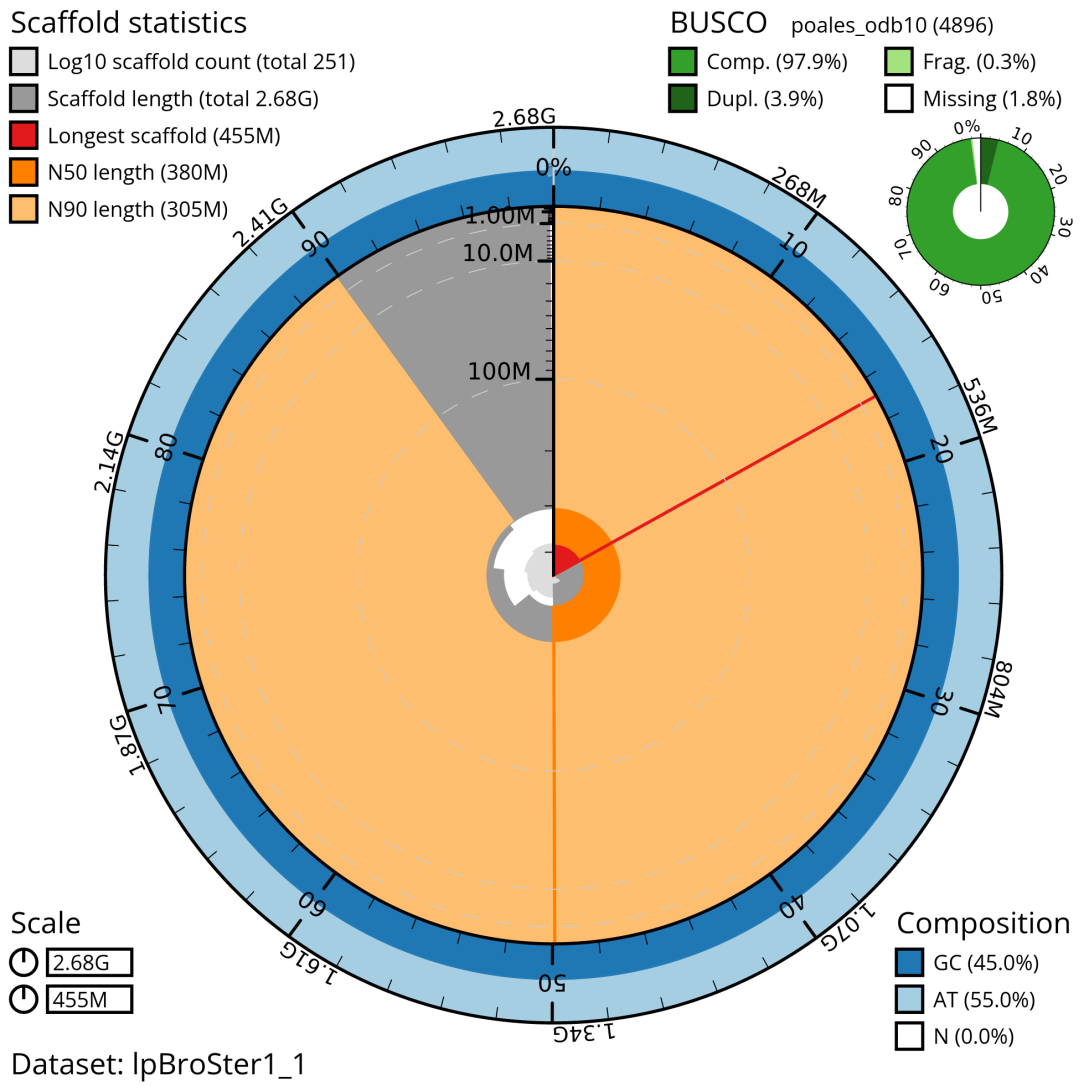
Genome assembly of *Bromus sterilis*, lpBroSter1.1: metrics. The BlobToolKit Snailplot shows N50 metrics and BUSCO gene completeness. The main plot is divided into 1,000 size-ordered bins around the circumference with each bin representing 0.1% of the 2,678,566,086 bp assembly. The distribution of scaffold lengths is shown in dark grey with the plot radius scaled to the longest scaffold present in the assembly (454,733,196 bp, shown in red). Orange and pale-orange arcs show the N50 and N90 scaffold lengths (379,526,086 and 304,514,070 bp), respectively. The pale grey spiral shows the cumulative scaffold count on a log scale with white scale lines showing successive orders of magnitude. The blue and pale-blue area around the outside of the plot shows the distribution of GC, AT and N percentages in the same bins as the inner plot. A summary of complete, fragmented, duplicated and missing BUSCO genes in the poales_odb10 set is shown in the top right. An interactive version of this figure is available at https://blobtoolkit.genomehubs.org/view/lpBroSter1_1/dataset/lpBroSter1_1/snail.

**Figure 3.**
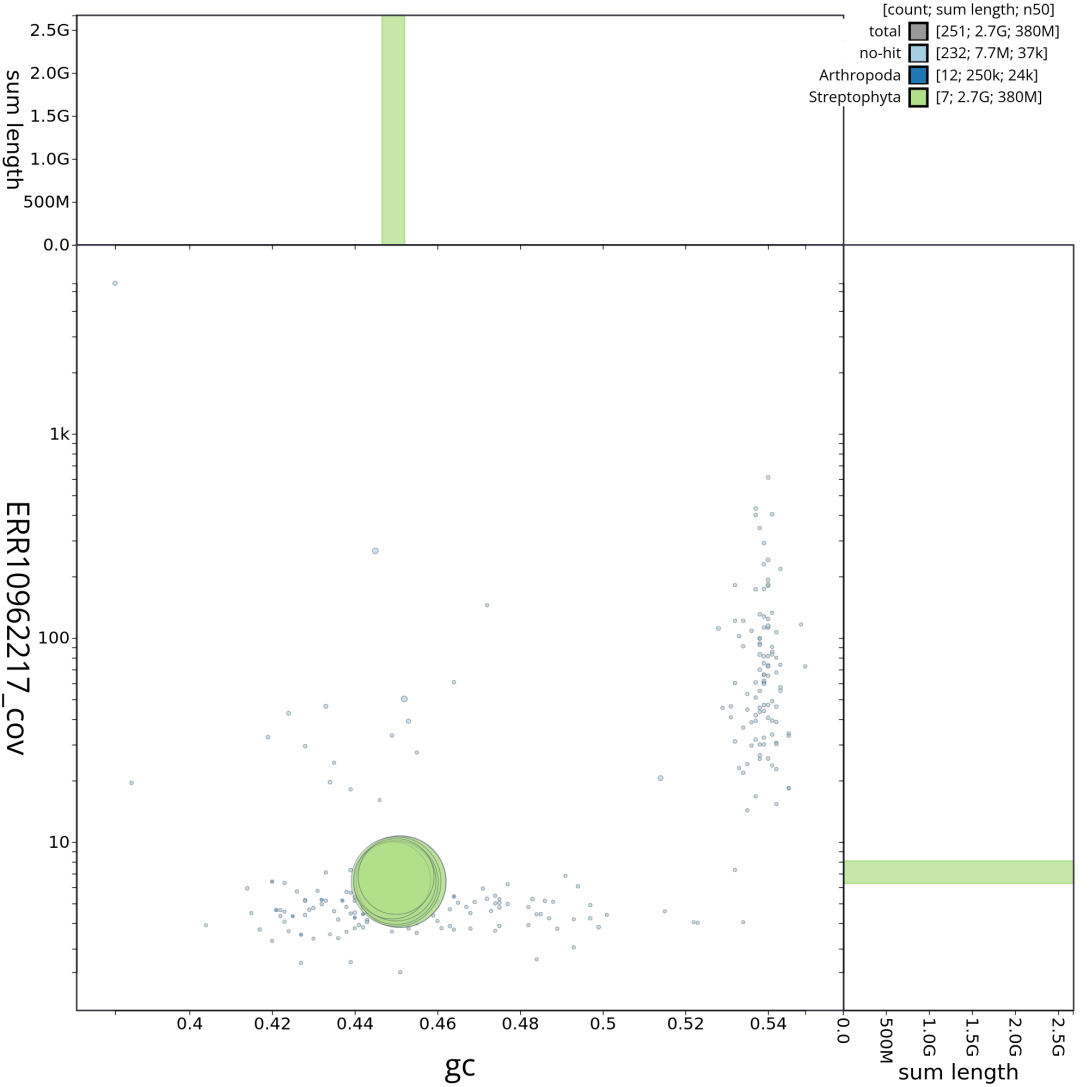
Genome assembly of *Bromus sterilis*, lpBroSter1.1: BlobToolKit GC-coverage plot. Scaffolds are coloured by phylum. Circles are sized in proportion to scaffold length. Histograms show the distribution of scaffold length sum along each axis. An interactive version of this figure is available at https://blobtoolkit.genomehubs.org/view/lpBroSter1_1/dataset/lpBroSter1_1/blob.

**Figure 4.**
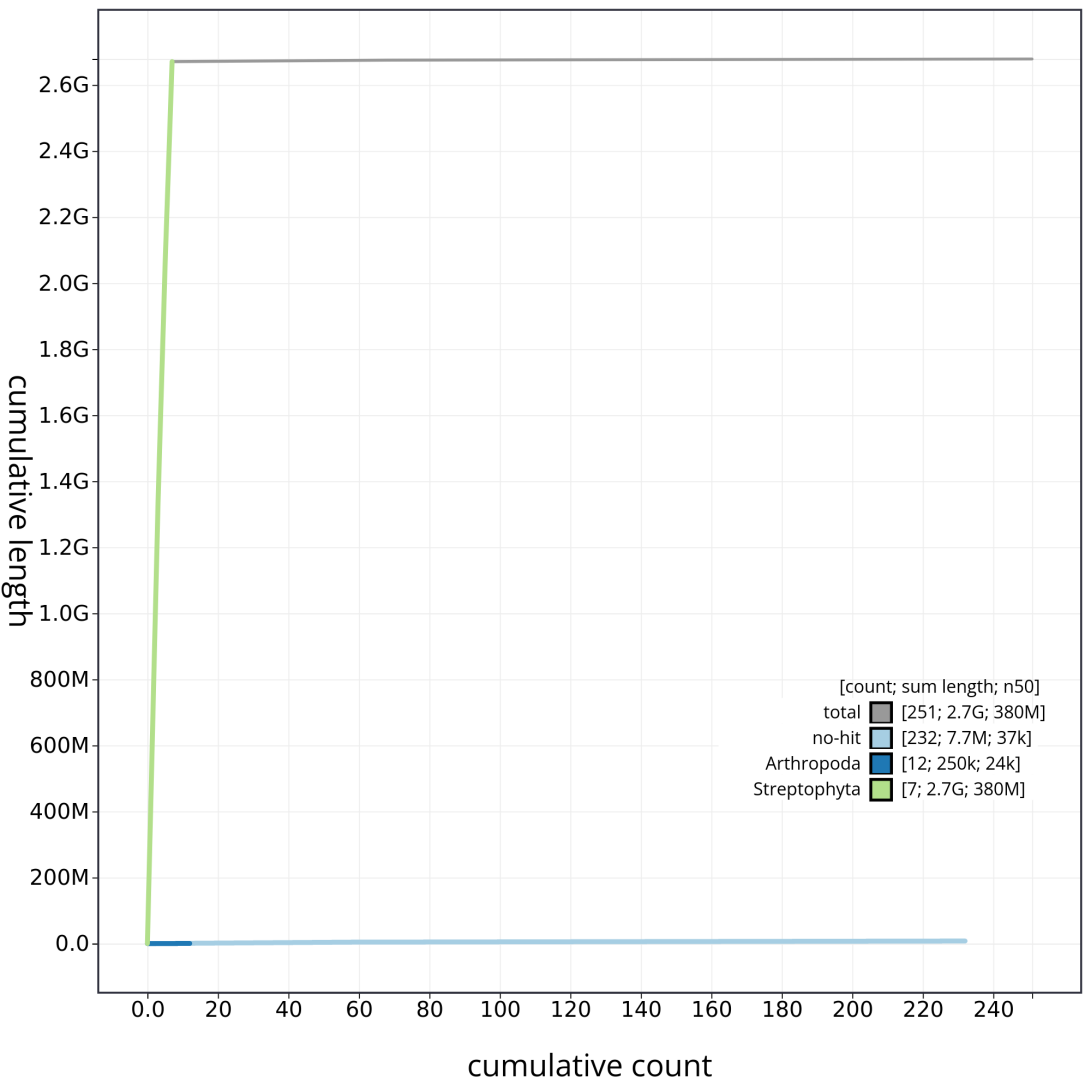
Genome assembly of *Bromus sterilis*, lpBroSter1.1: BlobToolKit cumulative sequence plot. The grey line shows cumulative length for all scaffolds. Coloured lines show cumulative lengths of scaffolds assigned to each phylum using the buscogenes taxrule. An interactive version of this figure is available at https://blobtoolkit.genomehubs.org/view/lpBroSter1_1/dataset/lpBroSter1_1/cumulative.

**Figure 5.**
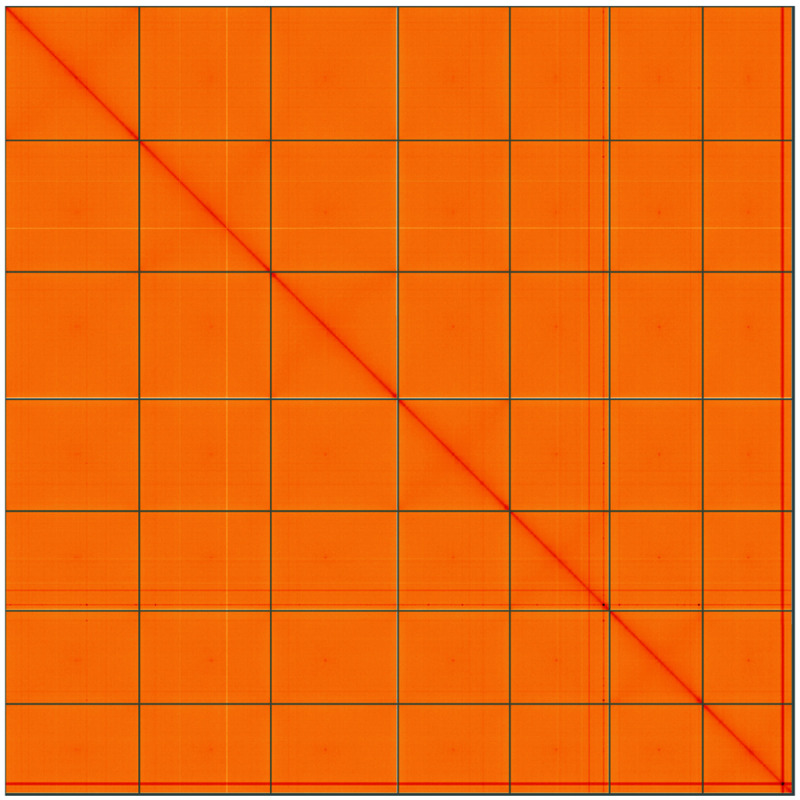
Genome assembly of *Bromus sterilis*, lpBroSter1.1: Hi-C contact map of the lpBroSter1.1 assembly, visualised using HiGlass. Chromosomes are shown in order of size from left to right and top to bottom. An interactive version of this figure may be viewed at https://genome-note-higlass.tol.sanger.ac.uk/l/?d=ZIoPopyHQRGTAw1AaXaJPg.

Manual assembly curation corrected 109 missing joins or mis-joins, reducing the scaffold number by 28.69%, and decreasing the scaffold N50 by 11.85%. The final assembly has a total length of 2,677.90 Mb in 249 sequence scaffolds with a scaffold N50 of 379.5 Mb ( Table 2) with 110 gaps. The snail plot in Figure 2 provides a summary of the assembly statistics, while the distribution of assembly scaffolds on GC proportion and coverage is shown in Figure 3. The cumulative assembly plot in Figure 4 shows curves for subsets of scaffolds assigned to different phyla. Most (99.72%) of the assembly sequence was assigned to 7 chromosomal-level scaffolds. Chromosome-scale scaffolds confirmed by the Hi-C data are named in order of size ( Figure 5; Table 3). The order and orientation of contigs on chromosome 7 between 269 Mb and 275 Mb are unclear. While not fully phased, the assembly deposited is of one haplotype. Contigs corresponding to the second haplotype have also been deposited. The mitochondrial and plastid genomes were also assembled and can be found as contigs within the multifasta file of the genome submission.

**Table 1.** Specimen and sequencing data for *Bromus sterilis*.

Project information			
**Study title**	Bromus sterilis (barren brome)		
**Umbrella BioProject**	PRJEB60321		
**Species**	*Bromus sterilis*		
**BioSample**	SAMEA9143060		
**NCBI taxonomy ID**	55777		
Specimen information			
**Technology**	**ToLID**	**BioSample** **accession**	**Organism part**
**PacBio long read sequencing**	lpBroSter1	SAMEA9143820	leaf
**Hi-C sequencing**	lpBroSter1	SAMEA9143819	leaf
**RNA sequencing**	lpBroSter1	SAMEA9143820	leaf
Sequencing information			
**Platform**	**Run accession**	**Read count**	**Base count (Gb)**
**Hi-C Illumina NovaSeq 6000**	ERR10968301	3.31e+09	499.93
**PacBio Sequel IIe**	ERR10962215	1.18e+06	10.6
**PacBio Sequel IIe**	ERR10962216	2.06e+06	23.08
**PacBio Sequel IIe**	ERR10962218	1.40e+06	12.54
**PacBio Sequel IIe**	ERR10962219	1.49e+06	13.64
**PacBio Sequel IIe**	ERR10962217	2.30e+06	18.92
**RNA Illumina NovaSeq 6000**	ERR10968302	5.87e+07	8.87

**Table 2.** Genome assembly data for *Bromus sterilis*, lpBroSter1.1.

Genome assembly		
Assembly name	lpBroSter1.1	
Assembly accession	GCA_950022295.1	
*Accession of alternate* *haplotype*	*GCA_950022485.1*	
Span (Mb)	2,677.90	
Number of contigs	361	
Contig N50 length (Mb)	338.4	
Number of scaffolds	249	
Scaffold N50 length (Mb)	379.5	
Longest scaffold (Mb)	454.73	
Assembly metrics *		*Benchmark*
Consensus quality (QV)	72.8	*≥ 50*
*k*-mer completeness	100.0%	*≥ 95%*
BUSCO **	C:97.9%[S:94.0%,D:3.9%],F:0.3%, M:1.8%,n:4,896	*C ≥ 95%*
Percentage of assembly mapped to chromosomes	99.72%	*≥ 95%*
Organelles	Mitochondrial genome: 523.28 kb; plastid genome: 136.96 kb	*complete single alleles*
Genome annotation at Ensembl		
Number of protein-coding genes	29,147	
Number of non-coding genes	12,954	
Number of gene transcripts	53,354	

* Assembly metric benchmarks are adapted from column VGP-2020 of “Table 1: Proposed standards and metrics for defining genome assembly quality” from Rhie *et al.* (2021). ** BUSCO scores based on the poales_odb10 BUSCO set using version 5.4.3. C = complete [S = single copy, D = duplicated], F = fragmented, M = missing, n = number of orthologues in comparison. A full set of BUSCO scores is available at https://blobtoolkit.genomehubs.org/view/lpBroSter1_1/dataset/lpBroSter1_1/busco.

**Table 3.** Chromosomal pseudomolecules in the genome assembly of *Bromus sterilis*, lpBroSter1.

INSDC accession	Name	Length (Mb)	GC%
OX465595.1	1	454.73	45.0
OX465596.1	2	446.1	45.0
OX465597.1	3	431.55	45.0
OX465598.1	4	379.53	45.0
OX465599.1	5	338.38	45.0
OX465600.1	6	315.78	45.0
OX465601.1	7	304.51	45.0
OX465602.1	MT	0.52	44.5
OX465603.1	Pltd	0.14	38.0

The estimated Quality Value (QV) of the final assembly is 72.8 with *k*-mer completeness of 100.0%, and the assembly has a BUSCO v5.4.3 completeness of 97.9% (single = 94.0%, duplicated = 3.9%), using the poales_odb10 reference set ( *n* = 4,896).

Metadata for specimens, BOLD barcode results, spectra estimates, sequencing runs, contaminants and pre-curation assembly statistics are given at https://links.tol.sanger.ac.uk/species/55777.

## Genome annotation report

The *Bromus sterilis* genome assembly (GCA_950022295.1) was annotated at the European Bioinformatics Institute (EBI) on Ensembl Rapid Release. The resulting annotation includes 53,354 transcribed mRNAs from 29,147 protein-coding and 12,954 non-coding genes ( Table 2; https://rapid.ensembl.org/Bromus_sterilis_GCA_950022295.1/Info/Index). The average transcript length is 2,668.62. There are 1.27 coding transcripts per gene and 4.14 exons per transcript.

## Methods

### Sample acquisition, DNA barcoding and genome size estimation

A specimen of *Bromus sterilis* (specimen ID KDTOL10166, ToLID lpBroSter1) was collected from Royal Botanic Gardens Kew, Richmond, Surrey, UK (latitude 51.47, longitude –0.30) on 2021-04-11. The specimen was collected and identified by Maarten J. M. Christenhusz (Royal Botanic Gardens Kew) and frozen at –80 °C. The herbarium voucher associated with the sequenced plant is K001527105 and is deposited in the herbarium of RBG Kew (K).

The initial species identification was verified by an additional DNA barcoding process according to the framework developed by Twyford *et al*. (2024). Part of the plant specimen was preserved in silica gel desiccant. A DNA extraction from the dried plant was amplified by PCR for standard barcode markers, with the amplicons sequenced and compared to public sequence databases including GenBank and the Barcode of Life Database (BOLD). The barcode sequences for this specimen are openly available on BOLD ( Ratnasingham & Hebert, 2007). Following whole genome sequence generation, DNA barcodes were also used alongside the initial barcoding data for sample tracking through the genome production pipeline at the Wellcome Sanger Institute ( Twyford *et al.*, 2024). The standard operating procedures for the Darwin Tree of Life barcoding have been deposited on protocols.io ( Beasley *et al.*, 2023).

The genome size was estimated by flow cytometry at the Royal Botanic Gardens Kew using the fluorochrome propidium iodide and following the ‘one-step’ method as outlined in Pellicer *et al.* (2021). For this species, the General Purpose Buffer (GPB) supplemented with 3% PVP and 0.08% (v/v) beta-mercaptoethanol was used for isolation of nuclei ( Loureiro *et al.*, 2007), and the internal calibration standard was *Pisum sativum* ‘Ctirad’ with an assumed 1C-value of 4,445 Mb ( Doležel *et al.*, 1998).

### Nucleic acid extraction

The workflow for high molecular weight (HMW) DNA extraction at the WSI Tree of Life Core Laboratory includes a sequence of core procedures: sample preparation; sample homogenisation, DNA extraction, fragmentation, and clean-up. In sample preparation, the lpBroSter1 sample was weighed and dissected on dry ice ( Jay *et al.*, 2023). For sample homogenisation, leaf tissue was cryogenically disrupted using the Covaris cryoPREP ^®^ Automated Dry Pulverizer ( Narváez-Gómez *et al.*, 2023).

HMW DNA was extracted using the Automated Plant MagAttract v2 protocol ( Todorovic *et al.*, 2023). HMW DNA was sheared into an average fragment size of 12–20 kb in a Megaruptor 3 system ( Bates *et al.*, 2023). Sheared DNA was purified by solid-phase reversible immobilisation, using AMPure PB beads to eliminate shorter fragments and concentrate the DNA ( Strickland *et al.*, 2023). The concentration of the sheared and purified DNA was assessed using a Nanodrop spectrophotometer and Qubit Fluorometer and Qubit dsDNA High Sensitivity Assay kit. Fragment size distribution was evaluated by running the sample on the FemtoPulse system.

RNA was extracted from leaf tissue of lpBroSter1 in the Tree of Life Laboratory at the WSI using the RNA Extraction: Automated MagMax™ *mir*Vana protocol ( do Amaral *et al.*, 2023). The RNA concentration was assessed using a Nanodrop spectrophotometer and a Qubit Fluorometer using the Qubit RNA Broad-Range Assay kit. Analysis of the integrity of the RNA was done using the Agilent RNA 6000 Pico Kit and Eukaryotic Total RNA assay.

Protocols developed by the WSI Tree of Life core laboratory are publicly available on protocols.io ( Denton *et al.*, 2023).

### Sequencing

Pacific Biosciences HiFi circular consensus DNA sequencing libraries were constructed according to the manufacturers’ instructions. Poly(A) RNA-Seq libraries were constructed using the NEB Ultra II RNA Library Prep kit. DNA and RNA sequencing was performed by the Scientific Operations core at the WSI on Pacific Biosciences Sequel IIe (HiFi) and Illumina NovaSeq 6000 (RNA-Seq) instruments. Hi-C data were also generated from leaf tissue of lpBroSter1 using the Arima-HiC v2 kit. The Hi-C sequencing was performed using paired-end sequencing with a read length of 150 bp on the Illumina NovaSeq 6000 instrument.

### Genome assembly, curation and evaluation

***Assembly***

The HiFi reads were first assembled using Hifiasm ( Cheng *et al.*, 2021) with the --primary option. Haplotypic duplications were identified and removed using purge_dups ( Guan *et al.*, 2020). The Hi-C reads were mapped to the primary contigs using bwa-mem2 ( Vasimuddin *et al.*, 2019). The contigs were further scaffolded using the provided Hi-C data ( Rao *et al.*, 2014) in YaHS ( Zhou *et al.*, 2023) using the --break option. The scaffolded assemblies were evaluated using Gfastats ( Formenti *et al.*, 2022), BUSCO ( Manni *et al.*, 2021) and MERQURY.FK ( Rhie *et al.*, 2020). The organelle genomes were assembled using OATK ( Zhou, 2023).

***Curation***

The assembly was decontaminated using the Assembly Screen for Cobionts and Contaminants (ASCC) pipeline (article in preparation). Manual curation was primarily conducted using PretextView ( Harry, 2022), with additional insights provided by JBrowse2 ( Diesh *et al.*, 2023) and HiGlass ( Kerpedjiev *et al.*, 2018). Scaffolds were visually inspected and corrected as described by Howe *et al*. (2021). Any identified contamination, missed joins, and mis-joins were corrected, and duplicate sequences were tagged and removed. The process is documented at https://gitlab.com/wtsi-grit/rapid-curation (article in preparation).

***Evaluation of final assembly***

A Hi-C map for the final assembly was produced using bwa-mem2 ( Vasimuddin *et al.*, 2019) in the Cooler file format ( Abdennur & Mirny, 2020). To assess the assembly metrics, the *k*-mer completeness and QV consensus quality values were calculated in Merqury ( Rhie *et al.*, 2020). This work was done using the “sanger-tol/readmapping” ( Surana *et al.*, 2023a) and “sanger-tol/genomenote” ( Surana *et al.*, 2023b) pipelines. The genome readmapping pipelines were developed using the nf-core tooling ( Ewels *et al.*, 2020), use MultiQC ( Ewels *et al.*, 2016), and make extensive use of the Conda package manager, the Bioconda initiative ( Grüning *et al.*, 2018), the Biocontainers infrastructure ( da Veiga Leprevost *et al.*, 2017), and the Docker ( Merkel, 2014) and Singularity ( Kurtzer *et al.*, 2017) containerisation solutions. The genome was also analysed within the BlobToolKit environment ( Challis *et al.*, 2020) and BUSCO scores ( Manni *et al.*, 2021) were calculated.

Table 4 contains a list of relevant software tool versions and sources.

**Table 4.** Software tools: versions and sources.

Software tool	Version	Source
BlobToolKit	4.2.1	https://github.com/blobtoolkit/blobtoolkit
BUSCO	5.3.2	https://gitlab.com/ezlab/busco
bwa-mem2	2.2.1	https://github.com/bwa-mem2/bwa-mem2
Cooler	0.8.11	https://github.com/open2c/cooler
Gfastats	1.3.6	https://github.com/vgl-hub/gfastats
Hifiasm	0.16.1-r375	https://github.com/chhylp123/hifiasm
HiGlass	1.11.6	https://github.com/higlass/higlass
Merqury	MerquryFK	https://github.com/thegenemyers/MERQURY.FK
MitoHiFi	2	https://github.com/marcelauliano/MitoHiFi
OATK	0.1	https://github.com/c-zhou/oatk
PretextView	0.2	https://github.com/wtsi-hpag/PretextView
purge_dups	1.2.3	https://github.com/dfguan/purge_dups
sanger-tol/genomenote	v1.0	https://github.com/sanger-tol/genomenote
sanger-tol/readmapping	1.1.0	https://github.com/sanger-tol/readmapping/tree/1.1.0
YaHS	1.2a	https://github.com/c-zhou/yahs

### Wellcome Sanger Institute – Legal and Governance

The materials that have contributed to this genome note have been supplied by a Darwin Tree of Life Partner. The submission of materials by a Darwin Tree of Life Partner is subject to the **‘Darwin Tree of Life Project Sampling Code of Practice’**, which can be found in full on the Darwin Tree of Life website here. By agreeing with and signing up to the Sampling Code of Practice, the Darwin Tree of Life Partner agrees they will meet the legal and ethical requirements and standards set out within this document in respect of all samples acquired for, and supplied to, the Darwin Tree of Life Project.

Further, the Wellcome Sanger Institute employs a process whereby due diligence is carried out proportionate to the nature of the materials themselves, and the circumstances under which they have been/are to be collected and provided for use. The purpose of this is to address and mitigate any potential legal and/or ethical implications of receipt and use of the materials as part of the research project, and to ensure that in doing so we align with best practice wherever possible. The overarching areas of consideration are:

•     Ethical review of provenance and sourcing of the material

•     Legality of collection, transfer and use (national and international) 

Each transfer of samples is further undertaken according to a Research Collaboration Agreement or Material Transfer Agreement entered into by the Darwin Tree of Life Partner, Genome Research Limited (operating as the Wellcome Sanger Institute), and in some circumstances other Darwin Tree of Life collaborators.

## Data Availability

European Nucleotide Archive: *Bromus sterilis* (barren brome). Accession number PRJEB60321; https://identifiers.org/ena.embl/PRJEB60321 ( Wellcome Sanger Institute, 2023). The genome sequence is released openly for reuse. The *Bromus sterilis* genome sequencing initiative is part of the Darwin Tree of Life (DToL) project. All raw sequence data and the assembly have been deposited in INSDC databases. Raw data and assembly accession identifiers are reported in Table 1.

## Author information

Members of the Royal Botanic Gardens Kew Genome Acquisition Lab are listed here: https://doi.org/10.5281/zenodo.12625079.

Members of the Plant Genome Sizing collective are listed here: https://doi.org/10.5281/zenodo.7994306.

Members of the Darwin Tree of Life Barcoding collective are listed here: https://doi.org/10.5281/zenodo.12158331.

Members of the Wellcome Sanger Institute Tree of Life Management, Samples and Laboratory team are listed here: https://doi.org/10.5281/zenodo.12162482.

Members of Wellcome Sanger Institute Scientific Operations: Sequencing Operations are listed here: https://doi.org/10.5281/zenodo.12165051.

Members of the Wellcome Sanger Institute Tree of Life Core Informatics team are listed here: https://doi.org/10.5281/zenodo.12160324.

Members of the Tree of Life Core Informatics collective are listed here: https://doi.org/10.5281/zenodo.12205391.

Members of the Darwin Tree of Life Consortium are listed here: https://doi.org/10.5281/zenodo.4783558.
