# Supplementary material for: The genome sequence of barren brome, Bromus sterilis L. (Poaceae)
Source: Wellcome Open Res. 2024 Nov 20;9:534. Originally published 2024 Sep 18. [Version 2] doi: 10.12688/wellcomeopenres.22994.2 (PMC11462126; doi:10.12688/wellcomeopenres.22994.2)
Supplement: Peer review history

# Reviewer report: Reviewer response for version 2

**DOI:** 10.21956/wellcomeopenres.25626.r115911

**Published:** 2024-12-30

**Authors:** Ajoy Kumar Roy

**Affiliations:** 1 ICAR-Indian Grassland and Fodder Research Institute, Jhansi, India

The paper provides genome assembly of grass Bromus sterilis. The paper is well written with necessary details of methodology followed as well as results and analysis. The chromosomal as well as cytoplasmic organelle like plastids and mitochondria were also considered. 

 The gene annotation also identified the protein coding genes. The paper is informative and should be Indexed.

Are sufficient details of methods and materials provided to allow replication by others?

Yes

Is the rationale for creating the dataset(s) clearly described?

Yes

Are the datasets clearly presented in a useable and accessible format?

Yes

Are the protocols appropriate and is the work technically sound?

Yes

Reviewer Expertise:

genetics and biotechnology especially of forage crops

I confirm that I have read this submission and believe that I have an appropriate level of expertise to confirm that it is of an acceptable scientific standard.

# Reviewer report: Reviewer response for version 2

**DOI:** 10.21956/wellcomeopenres.25626.r115908

**Published:** 2024-12-27

**Authors:** Eduardo Cires

**Affiliations:** 1 University of Oviedo, Oviedo, Spain

The study represents a significant scientific breakthrough by sequencing the whole genome of *Bromus sterilis* (Poaceae), a diploid grass species known as a harmful weed in cereal crops. This achievement provides a high-quality, chromosome-level genome assembly, which is supported by excellent results from various evaluation methods. The genome annotation, identifying 29,147 protein-coding genes, adds valuable depth to this resource.

 The manuscript is well-structured, with a concise, clear, and comprehensive description of the species, methodology, and results. The chosen methods are appropriate, the illustrations of the results are accurate and informative, and the completeness of the genome assembly is satisfactory.

 This genomic resource holds great promise for advancing our understanding of grass genome evolution and offers practical applications for developing strategies to manage Bromus sterilis in agricultural systems. I find no shortcomings in this work and recommend its acceptance in its current form.

Are sufficient details of methods and materials provided to allow replication by others?

Yes

Is the rationale for creating the dataset(s) clearly described?

Yes

Are the datasets clearly presented in a useable and accessible format?

Yes

Are the protocols appropriate and is the work technically sound?

Yes

Reviewer Expertise:

Plant Evolution

I confirm that I have read this submission and believe that I have an appropriate level of expertise to confirm that it is of an acceptable scientific standard.

# Reviewer report: Reviewer response for version 2

**DOI:** 10.21956/wellcomeopenres.25626.r112699

**Published:** 2024-12-09

**Authors:** Katarzyna Krawczyk

**Affiliations:** 1 University of Warmia and Mazury in Olsztyn, Olsztyn, Poland

The authors present in the manuscript data concerning the genome of *Bromus sterilis*, a diploid species of grass that is a weed in cereal crops. The description of the species, methods and results is concise, clear and comprehensive. The completeness of the genome is satisfactory. The methods are well chosen, the results are correctly illustrated. I have no comments on this work and recommend its acceptance in its current form.

Are sufficient details of methods and materials provided to allow replication by others?

Yes

Is the rationale for creating the dataset(s) clearly described?

Yes

Are the datasets clearly presented in a useable and accessible format?

Yes

Are the protocols appropriate and is the work technically sound?

Yes

Reviewer Expertise:

Phylogenomics, comparative genomics, transcriptomics

I confirm that I have read this submission and believe that I have an appropriate level of expertise to confirm that it is of an acceptable scientific standard.

# Reviewer report: Reviewer response for version 1

**DOI:** 10.21956/wellcomeopenres.25321.r100193

**Published:** 2024-10-08

**Authors:** Jesse R. Lasky

**Affiliations:** 1 The Pennsylvania State University - University Park Campus, University Park, Pennsylvania, USA

The authors present a genome assembly for Bromus sterilis. The assembly appears to be of relatively high quality. My one comment is minor, the authors state that sterilis is "strictly inbreeding" however this is not true if, as they state, and I believe likely, sterilis "occasionally outcross".  I would instead say they are primarily selfing and largely inbred.

Are sufficient details of methods and materials provided to allow replication by others?

Yes

Is the rationale for creating the dataset(s) clearly described?

Yes

Are the datasets clearly presented in a useable and accessible format?

Yes

Are the protocols appropriate and is the work technically sound?

Yes

Reviewer Expertise:

Evolutionary genetics and physiology

I confirm that I have read this submission and believe that I have an appropriate level of expertise to confirm that it is of an acceptable scientific standard.

# Reviewer report: Reviewer response for version 1

**DOI:** 10.21956/wellcomeopenres.25321.r100188

**Published:** 2024-10-04

**Authors:** Peng-Fei Ma

**Affiliations:** 1 Germplasm Bank of Wild Species & Yunnan Key Laboratory of Crop Wild Relatives Omics, Kunming Institute of Botany, Chinese Academy of Sciences, Kunming, China

In this study, the authors sequenced the whole genome of an important grass species, a crop weed harmful to the yield. They obtained a high-quality and chromosome level genome assembly,  as indicated by the good results of multiple evaluation approaches. They also completed the genome annotation with 29,147 protein-coding genes. This will become valuable genomic resources for studying the grass genome evolution in general and helpful for controlling this weed in particular. There is one error in the manuscript 'genome sequence report' and the total HiFi data generated is not 18.92 Gb, and this is just output from one of the PacBio sequencing run (see Table 1).

Are sufficient details of methods and materials provided to allow replication by others?

Yes

Is the rationale for creating the dataset(s) clearly described?

Yes

Are the datasets clearly presented in a useable and accessible format?

Yes

Are the protocols appropriate and is the work technically sound?

Yes

Reviewer Expertise:

Plant phylogeny, polyploidy and genome evolution.

I confirm that I have read this submission and believe that I have an appropriate level of expertise to confirm that it is of an acceptable scientific standard.
